# Harnessing the Potential of Google Searches for Understanding Dynamics of Intimate Partner Violence Before and After the COVID-19 Outbreak

**DOI:** 10.1007/s10680-022-09619-2

**Published:** 2022-05-30

**Authors:** Selin Köksal, Luca Maria Pesando, Valentina Rotondi, Ebru Şanlıtürk

**Affiliations:** 1 Department of Social and Political Sciences, Bocconi University, Via Rontgen 1, 20136 Milan, Italy; 2 Department of Sociology and Centre on Population Dynamics, McGill University, 855 Sherbrooke Street West, Montréal, Québec H3A 2T7 Canada; 3 University of Applied Sciences and Arts of Italian Switzerland, Via Pobiette 11, 6928 Manno, Switzerland; 4 Leverhulme Centre for Demographic Science, Nuffield College, University of Oxford, Oxford, UK; 5 Max Planck Institute for Demographic Research, Konrad-Zuse Str. 1, 18057 Rostock, Germany

**Keywords:** Digital data, Google Trends, Intimate partner violence, Facebook survey, Italy, COVID-19

## Abstract

Most social phenomena are inherently complex and hard to measure, often due to under-reporting, stigma, social desirability bias, and rapidly changing external circumstances. This is for instance the case of Intimate Partner Violence (IPV), a highly-prevalent social phenomenon which has drastically risen in the wake of the COVID-19 pandemic. This paper explores whether big data—an increasingly common tool to track, nowcast, and forecast social phenomena in close-to-real time—might help track and understand IPV dynamics. We leverage online data from Google Trends to explore whether online searches might help reach “hard-to-reach” populations such as victims of IPV using Italy as a case-study. We ask the following questions: Can digital traces help predict instances of IPV—both potential threat and actual violent cases—in Italy? Is their predictive power weaker or stronger in the aftermath of crises such as COVID-19? Our results suggest that online searches using selected keywords measuring different facets of IPV are a powerful tool to track *potential* threats of IPV before and during global-level crises such as the current COVID-19 pandemic, with stronger predictive power post outbreaks. Conversely, online searches help predict *actual* violence only in post-outbreak scenarios. Our findings, validated by a Facebook survey, also highlight the important role that socioeconomic status (SES) plays in shaping online search behavior, thus shedding new light on the role played by *third-level* digital divides in determining the predictive power of digital traces. More specifically, they suggest that forecasting might be more reliable among high-SES population strata.

**Supplementary Information:**

The online version contains supplementary material available at 10.1007/s10680-022-09619-2.

## Introduction

Social media and online platforms including Facebook, Twitter, Instagram, and Google are tools by which millions of people search, spread, share, and exchange information. As such, bits of information obtained through online platforms—also called “digital traces”—have increasingly become valuable data sources to address some of the most pressing social phenomena that we are confronted with every day (Lazer et al., 2020), providing great opportunities yet also raising statistical, computational, and ethical challenges (Cesare et al., 2018).

Digital-trace data hold huge and underappreciated potential for studying global social phenomena that are inherently hard to measure (e.g., due to stigma, under-reporting, or social desirability bias) not only due to the complexity and sensitivity of some topics, but also because they provide the *only* way of tracking changes in social phenomena and societal dynamics that occur in close-to-real time. While in some instances tracking societal changes in real time is not essential, this is not the case when dealing with pressing public-health concerns such as the very high (WHO, 2013)—and rising—prevalence of Intimate Partner Violence (IPV) worldwide (Lindberg et al., 2020; Peterman et al., 2020; Abel & McQueen, 2020; WHO, 2020; Arenas-Arroyo et al., 2021). In a situation of lockdown such as the one imposed by the COVID-19 pandemic, women’s ability to escape abusive situations within their houses and their ability to reach their support networks are significantly reduced. At the same time, confinement measures might increase consumption of alcohol and other substances, while the increased economic uncertainty due to the global health crisis might trigger additional emotional stress, all elements which are associated with the perpetration of IPV (Storey, 2020; Card & Dahl, 2011; Aizer, 2010, 2011; Schneider et al., 2016).

As tracking instances of IPV is challenging—and particularly so in times of crises during which reporting tends to be even lower than usual—this study addresses the question of whether online searches from Google Trends might help reach this “traditionally difficult-to-reach” population (Xue et al., 2019a) using Italy as a case study. One of the key advantages of using digital traces—which are generated from the use of digital technologies, rather than being based on reporting—is precisely that they can help address issues where reporting or social desirability biases that preclude systematic reporting are prevalent—such as IPV or abortion (Reis & Brownstein, 2010).

A focus on Google searches (rather than, for instance, Tweets) is valuable in a context of global crisis for two reasons. First, Google searches are fully anonymous (while data originated from Social Network Platforms are not) and widely used across population strata. Second, in a situation of strict lockdown, close interaction with partners, and limited independence, Google has likely been one unique way for women to look for information and seek help privately without having to speak up, especially when accessed through mobile devices such as smartphones. Supposing a woman faces *potential* threats of violence within the household and has limited information/knowledge on support systems in place, we hypothesize that she might turn to the Internet to gather information and resources before ultimately reaching out to the IPV helpline to seek help. Similarly, a woman facing an *actual* threat of violence might turn to the Internet to gather resources on the most immediate type of help available and connect to social-support systems (e.g., in the context of Italy, typing the anti-violence helpline number 1522, which also connects to an online chat), and ultimately call an ambulance if needed. As of February 2022, Google has also launched a new feature for individuals who are seeking information or help on IPV through displaying a box with direct access to the national domestic violence helpline.^1^ This is possible through individuals’ searches using IPV-related keywords, a feature which makes Google an increasingly effective source to seek help.

On a broader level, if such online information turns out to be a strong predictor of actual instances of violence, this would suggest that online searches might be a key—and, to date, underappreciated—resource for tracking IPV and getting a “fair” picture of the brunt of domestic violence that women bear daily.

The rationale for choosing Italy for a study of this kind is twofold. First, IPV is a highly gendered phenomenon and Italy remains a very gender-traditional country, with a rooted focus on familistic culture regarding the societal role of women (Lomazzi, 2017). Italy’s gender culture is oriented towards the cultural dominance of the male breadwinner model and a traditional division of work between men and women based on a complementarity gender-role model which shows very slow evidence of change (Naldini & Jurado, 2013; Santero & Naldini, 2020).^2^ High IPV prevalence in the country reflects broader unequal gender dynamics. For instance, female labor force participation rates in Italy are among the lowest in Europe and OECD countries—around 57% according to 2019 estimates from the International Labour Organization (ILO)—and the gap between men and women’s participation in the labor force is around 20 percentage points (Mancini, 2017). For women active in the labor market, the difference between average men’s and women’s hourly earnings is very large—about 16 percentage points—and has remained fairly constant since the mid-1990s (Del Bono & Vuri, 2011). Not least, time devoted to housework is disproportionately higher for women and time devoted to childcare by mothers is twice as high as time devoted by fathers (Menniti et al., 2015). Second, Italy was among the first and hardest-hit countries by the first wave of COVID-19. As such, it was one of the first contexts that announced and implemented a strict and nationwide lockdown. Third, on top of data at the national level, we have access to unique data from Lombardy, the most populous region in Italy with more than one-sixth of Italy’s population—making up for approximately the populations of Finland and Norway combined—and producing more than a fifth of Italy’s Gross Domestic Product (GDP). Lombardy is also key in this context as the first COVID-19 case was identified there, leading to massive spreads first in the region, and then elsewhere (for a better overview of how the COVID-19 pandemic unfolded in Italy, see Online Appendix, Section 1).

To summarize, the current study addresses the following three research questions and tests the related hypotheses: Can digital traces from online sources such as Google Trends help track/predict instances of IPV in Italy (*RQ1*)? Evidence on the role of big data in other domains of social life suggests that this might well be the case (*HP1*). Provided digital data can be of help, is their predictive power weaker, stronger, or unaltered in the wake of key macro-level discontinuities such as the current COVID-19 pandemic (*RQ2*)? We hypothesize a stronger predictive power of digital data in the aftermath of the COVID-19 outbreak. While actual reporting of IPV is lower, online connectivity during crises tends to be high, either due to rising unemployment, forced lockdowns at home, or both (*HP2*). Lastly, despite Google is commonly used across population strata and digital divides in access (*first-level* digital divide) and usage (*second-level* digital divide) are less of a concern in Italy, there might be important differences in terms of who benefits the most from being online and from targeted online search behavior (*third-level* digital divide).^3^ Do high- and low-SES individuals search for similar keywords? Do they reap similar benefits from searching IPV-related keywords on Google (*RQ3*)? We hypothesize that high-SES individuals might search for very specific—and more targeted—keywords and be able to more effectively convert such information into explicit action (e.g., making a call to the IPV helpline). This might suggest that digital-trace data might be more useful predictors within high-SES population strata, thus leading to more reliable forecasting within this group (*HP3*).

Findings from this paper will inform whether digital information can be used to track IPV for hard-to-reach populations in Italy and reveal whether big data might provide “real time” bits that help target more immediate policy interventions in situations in which IPV cases cannot be reported easily or quickly, such as nationwide lockdowns. At a deeper level, the study will inform whether big data may ultimately allow to devise a sort of tracking system that serves as a precursor or signal for anticipating increases in IPV. As Internet penetration expands and digital divides by gender narrow, findings from this study will also have broad applicability to low- and middle-income countries (LMICs) in the years to come.

## Background

### IPV in the Wake of COVID-19 and Existing Evidence from Big Data

At the onset of the pandemic, several media outlets pointed out the upsurge of IPV cases both in Italy^4^$$^{,}$$^5^ and in the rest of the world.^6^$$^{,}$$^7^ Moreover, the World Health Organization (WHO) and UN Women underlined the risk of IPV being intensified during lockdown periods as security, health, and economic concerns became more pronounced (Arenas-Arroyo et al., 2021). Findings from recent studies regarding the impact of lockdown measures on the incidence of IPV are aligned with these concerns, which are pervasive across countries (Every-Palmer et al., 2020; Piquero et al., 2020; Leslie & Wilson, 2020; Perez-Vincent et al., 2020; Bullinger et al., 2021; Hsu & Henke, 2021; Agüero, 2021; Henke & Hsu, 2022). Research from Italy exploring the effectiveness of the media campaign “*Libera Puoi*”^8^—“Free you Can”—provides evidence of the immediate increase in number of calls during the first weeks of the lockdown, which remained at high levels until May 2020 (Colagrossi et al., 2020). The study also documents the abrupt rise in Google search volumes of the keyword *1522* which occurred right after the launch of the campaign.^9^ This is reasonable, as searching 1522 on Google is not only a way for women to look for resources, but also to connect to an online chat which could serve as a private means to seek immediate help.

Online data, most specifically social media data, provide a relatively recent—and, to date, underappreciated—source to analyze and interpret human behavior, as well as to nowcast and forecast individual and societal-level outcomes as diverse as fertility (Billari et al., 2016; Billari & Zagheni, 2017; Rampazzo et al., 2018), migration (Alexander et al., 2022; Zagheni & Weber, 2012; Zagheni et al., 2017), health and mortality (Delpierre & Kelly-Irving, 2018; Öhman & Watson, 2019), gender dynamics (Fatehkia et al., 2018; Kashyap et al., 2020), and family instability (Compton, 2019). Epidemiology was one of the first disciplines to promote the use of big data for research purposes by analyzing online search data to nowcast and forecast outbreaks such as influenza (Ginsberg et al., 2009), chicken pox (Pelat et al., 2009), and salmonella (Brownstein et al., 2009). The use of big data in epidemiological research came to be referred as *infodemiology* or *infoveillance* (Eysenbach, 2009) and, as IPV/gender-based violence is as a primary public health issue, IPV received the attention of infodemiology studies as well.

Social media data emerge as an especially useful source as forums, groups, and social networks allow users to share their experiences and establish emotional support among victims of IPV. For instance, Twitter has increasingly been used as a medium in IPV research based on big data, employing various computational methods, using tweets including IPV-related keywords or hashtags as units of analysis. Studies show evidence that there is an active Twitter community on violence against women, which tends to engage in conversations (Xue et al., 2019a); this community also highlights oft-neglected forms of violence such as reproductive coercion (McCauley et al., 2018) and provides important information on awareness campaigns, as well as a support platform (Purohit et al., 2016; Xue et al., 2019b). IPV studies using data from Pinterest (Carlyle et al., 2019) and Instagram (Carlyle et al., 2021), with predominantly female and young-adult users, respectively, corroborate the idea that social media platforms involve an experience-based narrative on different forms of violence and thus provide a valuable tool for policy makers and advocacy groups.

To the best of our knowledge, while the literature is gradually expanding in terms of leveraging big data and machine-learning (ML) techniques to study IPV (Rodriguez et al., 2020), only few studies to date have leveraged data on online searches from Google Trends to track dynamics of IPV—a contribution we intend to strengthen with the current study. A few exceptions are worth noting. First is a study by Anderberg et al. (2021), who designed a domestic violence index based on Internet search behavior to explore the incidence of IPV during the COVID-19 pandemic in the Greater London area and in Los Angeles. Second is a study by Berniell and Facchini (2021), who also developed a Google search intensity index to compare the incidence of IPV across 11 countries, finding an increase in IPV search intensity after the lockdown by 30%, with larger effects as more people stayed at home. Not least, in the case of Brazil, online search data on *feminicide* were found to be positively associated with female homicide rates but not with the introduction of feminicide-related laws (Martins-Filho et al., 2018). With this study, we contribute to this blooming literature by focusing on a range of IPV-related keywords (rather than indices) and combining heterogeneous data sources on calls to helplines at different levels of geographical aggregation.

Digital traces from Google Trends provide a useful source of information as, being the most commonly used search engine, Google is more widely used than, e.g., Twitter, Pinterest or Instagram, thus providing an arguably less biased picture of socio-demographic phenomena under investigation. As a matter of example, as of December 2020 Google was the most popular search engine in Italy, with a 95.7% share of the search engine market compared to the 2.9% of Bing and the 0.81% of Yahoo!.^10^ As of June 2020, social media penetration in Italy stood at 58%, with the most popular social network remaining Facebook, with 36.9 million users, followed by Instagram (27.7 million users), Linkedin (18.6 million users), and Pinterest (16.7 million users). As for the same period, Twitter counted only 10 million users, TikTok 6.6 million users and Reddit 2.8 million users.^11^ Also, Google Trends provide a flexible tool to select and investigate a wide array of potential keywords measuring heterogeneous facets of IPV.

### Help-Seeking Processes in Cases of IPV and the Role of SES

Liang et al. (2005, p. 73) conceptualize the help-seeking process in cases of intimate partner violence in three steps, namely: (i) *problem recognition and definition*, (ii) *decision to seek help*, and (iii) *selection of a help provider*. Each step is influenced by the social context that individuals live in, which in turn interacts with individual-level aspects such as gender and socioeconomic status. For instance, having access to limited economic and legal resources renders women more vulnerable and less capable of recognizing a case of IPV or deem it intolerable. Moreover, acceptance and adherence to social norms upholding the viewpoint that IPV is a private matter may prevent victims of violence from looking for help. Furthermore, loss of privacy and issues of stigma can arise as potential indirect costs of seeking formal forms of help, which may eventually lead to under-reporting of cases (Murray et al., 2015). Empirically, it has been well-established by both qualitative and quantitative research that stigma surrounding experiences of violence has a detrimental impact on victims’ self-esteem and mental well-being, as well as it discourages them from seeking immediate support (Crowe et al., 2021). We claim in this study that the Internet—and Google in particular—may offer a medium to privately voice some of these concerns and collect relevant information. In this study, we expect online information to be particularly instrumental during steps (i) and (ii). While step (iii) may take longer time, we hypothesize that being better informed on (i) and (ii) will ultimately lead women to seek the right form of help in a more timely manner. Nonetheless, the way in which IPV-related stigma may disrupt help-seeking processes is stratified, as higher-SES women might feel less ashamed of their exposure to violence and therefore be more likely to disclose their own experiences (Sylaska & Edwards, 2014). Online searches can serve as a useful tool to indirectly mitigate such stigmatization and social desirability concerns by warranting the anonymity of the help-seeker.

## Data and Methods

### Data

This study combines data from various sources and tests the predictive power of online data in the Italian context by relating digital traces with IPV-related content to information on actual IPV calls to official helplines.^12^ In a simplified framework, digital data provide information on our *predictors* of interest, while data on actual IPV cases/calls provide information on our *outcomes* of study.^13^

Starting from *predictors*, online data are obtained from Google Trends. Google Trends provide normalized data on the frequency of Google searches for a given time period, query, and location. Data obtained from Google Trends do not reflect the actual number or volume, but rather the frequency of online searches on Google on a scale from 0 to 100. Thus, zero does not necessarily mean a complete lack of Google searches; rather, it means that the frequency of the searches for a given parameter does not meet the minimum threshold set by Google. Google Trends provide search frequency data on a *daily* basis if the requested time range is 90 days or less, on a *weekly* basis if the time range is between 90 days and 5 years, and on a *monthly* basis if the requested time period is longer than 5 years.

Google Trends data are obtained through the R package *gtrendsR* (Massicotte & Eddelbuettel, 2021) from 2013 to 2020 (more details below on the exact time frame per data source). For the core analyses, we selected nine keywords which—except for the main Italian helpline number 1522 emerging as highly relevant from Colagrossi et al. (2020)—can be considered common search queries related to the study of IPV in an international global context. To start with, we intentionally kept these search queries broad—abstracting, for instance, from specific Italian jargon or regional language peculiarities—to encompass words that are highly prevalent and recurring in the international literature on IPV. The selected keywords were: *1522* (the IPV helpline number in Italy), *abuse* (abuso), *home & abuse* (casa & abuso), *home & rape* (casa & stupro),^14^*feminicide* (femminicidio), *rape* (stupro), *domestic violence* (violenza domestica), *gender-based violence* (violenza di genere), and *sexual violence* (violenza sessuale).^15^

We obtained three different data sets from Google Trends, for these queries, for different time periods and locations, such that the unit of time matches the one pertaining to the official records (*outcomes*, defined later). The first data set consists of daily data for Italy as a whole for the period between March 1 - June 30 for five years, from 2016 to 2020. Second, we created a data set composed of monthly data for the period March 1, 2013 to June 30, 2020, for all regions of Italy.^16^ We calculated four-month moving averages of Google search inquiries for each keyword and for each region. This step was performed in order to make the data compatible with the yearly-aggregated number of helpline calls at the regional level. Lastly, the third data set consists of daily Google Trends data, for the period between January 1, 2018 up to May 31, 2020 only for the region of Lombardy, Italy. For additional details on Google Trends data and adjustments made, see Online Appendix, Section 2.

Moving to *outcomes*, we rely on three sources of data. First, we obtained the daily number of calls (valid calls) to the 1522 anti-violence helpline from the Equal Opportunity Department (Presidency of the Italian Council of Ministers) for Italy as a whole, daily from March 1 to June 30, from 2016 to 2020. The second source of data is the number of monthly anti-violence 1522 calls, collected for the period March and June (one trimester) between years 2013-2020 and aggregated at the regional level and yearly level, i.e., the average for the trimester becomes our yearly estimate, so we have one time point per year per region, which means 20 data points per year. These data are publicly available from the Italian National Institute of Statistics (ISTAT, henceforth) website.^17^ The third one are data from AREU (Azienda Regionale Emergenza Urgenza – Regional Agency for Emergency Urgency), which provides data on daily calls to the AREU emergency number in Lombardy (112) for every day of the year between January 1, 2018 and May 30, 2020, alongside the reason behind the emergency call. This additional variable (“reason”) helps us identify calls that were received by AREU for reasons that can be traced back to accident or violence-related purposes.^18^

As the first source of data (Equal Opportunity Department), data from AREU are daily, yet they pertain to Lombardy only—the first source provides daily information for Italy as a whole with no regional identifier, hence no possibility to conduct region-specific analyses—and they record a different outcome (all emergency calls versus 1522 calls). The second source of data (ISTAT) tracks the same outcome—1522 calls—yet data for Lombardy (plus all other regions) are yearly, rather than daily. Overall, data from AREU add value to the analysis for two main reasons. First, these data pertain specifically to Lombardy, the most populous region of Italy and the hardest-hit by the COVID-19 pandemic. Second, from a theoretical standpoint, emergency calls—i.e., calls to request an ambulance, mostly—measure realized or “*actual* violence,” while 1522 calls may also measure “*potential* threat” or potential risk of experiencing IPV, two different yet equally important facets of the phenomenon. As AREU data are restricted and were shared confidentially, we limited all analyses to match the time frame in AREU data, i.e., mid-2020. This also allows us to focus on changes in IPV-related dynamics following the first lockdown, a real sudden and unexpected “shock” that, arguably, affected people’s lives differently from subsequent confinements or restrictions. We also note here that it would be ideal to validate the use of Google searches as predictors of IPV using both information on calls to helplines—as we do in this study—and crime reports, as shown in some of the relevant literature (Bullinger et al., 2021; Hsu & Henke, 2021). Unfortunately, we do not have access to crime reports in this context, hence we only rely on the former outcomes, keeping in mind that calls may not always move in tandem with crime reports.

### Methods

We first estimate the model reported in Eq. (1) using Ordinary Least Squares (OLS) regression with standard errors robust to heteroskedasticity:

1 $$\begin{aligned} Y_{t} = GoogleSearch_{t} + \lambda _y + \varepsilon _{t} \end{aligned}$$

where $Y_{t}$ indicates the number of valid calls received by 1522 in day t and $GoogleSearch_{t}$ represents the frequency of Google inquiries for the aforementioned keywords in day *t*. Year fixed effects ($\lambda _y$) are included to allow for heterogeneity across different years. In the core of the text we stratify analyses by time period (overall, pre-COVID, and post-lockdown) and provide separate coefficients by keyword. Additional analyses include regressions estimated on the pooled sample with a dummy variable (*post*) assuming value of 1 after March the 10th, when Italy enforced the lockdown, and keyword*post interactions to test whether coefficients are statistically different pre- and post-lockdown (Online Appendix). Analyses including month fixed effects and clustering standard errors at the month level are also reported in the Online Appendix (Section 3) and show analogous—less conservative—results. To account for the potential time lapse between Google search and call to helplines, Google searches are lagged by one week, with contemporaneous analyses reported in the Online Appendix. Our use of lags in this study is aimed at capturing—to the extent possible—decision-making processes of women at the individual level. It is in fact reasonable to expect a woman who is facing a threat of violence to resort to the Internet to gather information and seek online support first, and then reach out to IPV helplines after a few days if threats persist. Also, as robustness checks, we re-estimate our models using Poisson regressions—less intuitive but arguably better-suited to count data—and we run one specification also including a time dummy corresponding to the implementation of the “Libera Puoi" campaign (April 15, 2020) to make sure that our results are not driven by such awareness-raising initiative. To further corroborate results, we conclude by running a placebo test using as explanatory variables two keywords which are expected to increase during lockdown (*Pizza home delivery* and *Zumba*), yet are arguably unrelated to valid calls received by the helpline number.^19^

As far as the regional-level analysis is concerned, to mitigate the potential endogeneity due to several confounding factors which are correlated with both Google searches and number of 1522 calls, we draw on a set of regional controls such as educational attainment, unemployment rate, and GDP per capita. These data are also obtained from ISTAT. We therefore estimate the following model (Eq. 2) using OLS regression with clustered standard errors at the regional level:

2 $$\begin{aligned} Y_{rt} = GoogleSearch_{rt} + X_{rt} + \lambda _y+ \varepsilon _{rt} \end{aligned}$$

where $Y_{rt}$ indicates violence outcome (1522 or emergency number calls) for region r and for year t, $GoogleSearch_{rt}$ represents the frequency of Google inquiries for the aforementioned keywords in region r and averaged for year t,^20^$X_{rt}$ are the set of regional control variables, $\lambda _y$ are year fixed effects, and $\varepsilon _{rt}$ is the error term. In line with the above, analyses are stratified by time period, yet here we only have one time point per year, hence the dummy for the year 2020 is our proxy for post-outbreak dynamics, as the months that are included in the average yearly estimate are March-June for every year. Number of calls and control variables are adjusted to the regional population.

Lastly, analyses with AREU data are conducted at the level of Lombardy, rather than Italy, following Eq. (1), i.e., including a weekly lag, accounting for year fixed-effects, and stratified by time period. As above, analyses including month fixed effects and clustering standard errors at the month level are reported in the Online Appendix (Section 3) and show analogous results. Table 1 summarizes our data sources, alongside the spatio-temporal coverage and the empirical strategy.

**Table 1:** Data sources, coverage, and empirical specifications

Data source	Geographical unit	Time unit	Controls	Model
Italian Equal Opportunity Department, valid 1522 calls	Italy	Daily	Year fixed effects	OLS. SE robust to heteroskedasticity
ISTAT, valid 1522 calls	Regions within Italy	Yearly	Educational attainment, Unemployment rate, GDP, Year fixed effects	OLS. SE clustered at the regional level
AREU, number of calls	Lombardy Only	Daily	Year fixed effects	OLS. SE robust to heteroskedasticity

ISTAT, The Italian National Institute of Statistics; AREU, Regional Agency for Emergency Urgency; FE, fixed effects; SE, standard errors. Robustness checks, including analyses on the pooled sample with a dummy for the post-lockdown period (or dummy for year = 2020 for data source two) and interaction terms are reported in the Online Appendix

## Results

### Daily Calls to the Helpline Number, Overall Italy

**Fig. 1:**
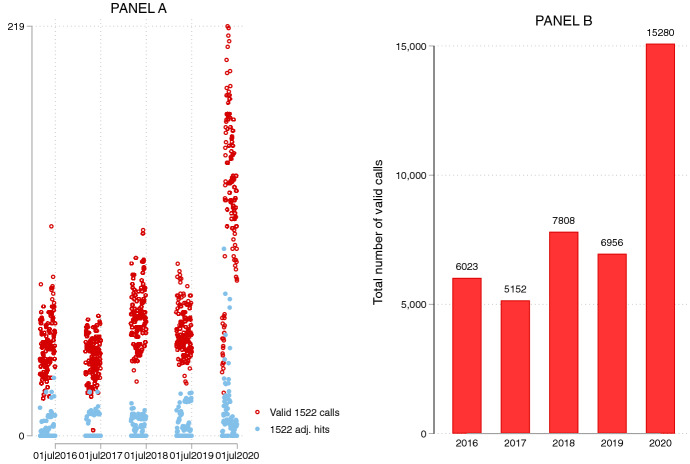
Daily number of valid 1522 calls and daily number of 1522 Google hits (**A**) and total number of 1522 calls (**B**) by year (over the period 1st of March-30th of June)

Panel A of Fig. 1 plots the daily number of valid 1522 calls and daily number of 1522 Google hits between March and July from 2016 to 2020. During the period between March and July 2020, the number of daily valid calls to the Italian helpline number increased considerably compared to the same period of the previous years. Moreover, and as shown also by Colagrossi et al. (2020), Google search volumes for the keyword 1522 rose considerably right after the Italian national lockdown and the launch of the “Libera Puoi" campaign. As a matter of comparison, during the first wave of the COVID-19 outbreak in Italy (between the 1st of March and the 30th of June 2020) there were 15,280 valid calls to 1522 (Panel B), +119.7% over the same period in 2019. Of these calls, 32% came from victims of violence or stalking seeking for help, 24% came from people seeking information about the helpline 1522, 6% came from people reporting violence. The remaining were related to general information seeking (37%)^21^ and emergency (1%).

**Fig. 2:**
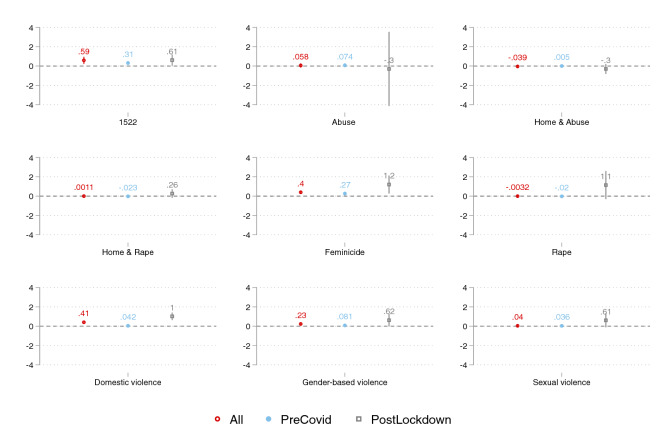
Coefficient plot from regressions of daily 1522 valid calls on Google searches, by selected keywords (whole Italy), lagged predictors

Figure 2 reports coefficient plots from regressions predicting daily 1522 valid calls, for Italy as a whole, as a function of our selected keywords. We provide results separately by keyword and report three estimates per panel: whole sample (empty dot), pre-COVID data (filled dot), and post-lockdown data (empty square). Google hits, i.e., the frequency of queries, for keywords *1522*, *feminicide*, *domestic violence*, and *gender-based violence* are consistently positively and significantly correlated with helpline calls across the whole time period (empty dot). Table A2 in the Online Appendix reports corresponding estimates for the whole period, which are also robust to including month fixed effects (Table A3) and clustered standard errors at the month level (Table A4). Overall, Google searches seem better predictive of helpline calls in the post-lockdown period, at least for the words *1522*, *home & rape*, *feminicide*, *rape*, *domestic violence*, *gender-based violence*, and *sexual violence*. Models on the pooled sample with a post-lockdown dummy interacted with each keyword (Table A5) show that for *domestic violence* and *feminicide* the association between searches and calls is significantly higher in the post-lockdown period yet—despite the consistently positive sign across models—no differential associations are observed for the remaining keywords. The sole dummy *post* also shows how significantly higher helpline calls were in the post-lockdown period. Appendix Figure A1 reports a similar panel of estimates, yet with contemporaneous (instead of lagged) predictors. Overall, evidence suggests that online searches are better at forecasting than nowcasting, in line with our theorized hypothesis above—hence the former estimates in the main analysis.

The main results are virtually unchanged when estimating a Poisson model (Table A6), or when adding a dummy for the Libera Puoi campaign (Table A7), thus suggesting that results are not driven by such awareness-raising initiative. Results from the placebo test (reported in Table A8) show that, as expected, the two keywords selected (*Zumba* and *Pizza home delivery*) are statistically unrelated to valid calls received by the 1522 helpline number and even exhibit opposite (hence, arguably “random”) signs.

### Monthly Calls to the Helpline Number, Regions Across Italy

**Fig. 3:**
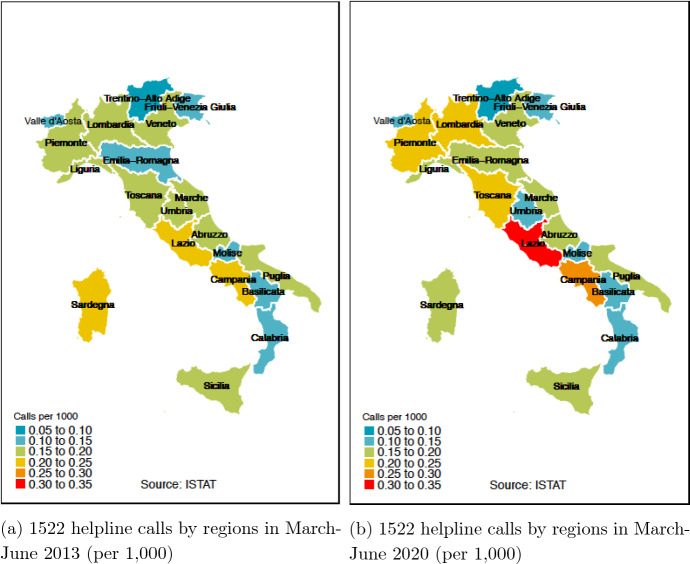
1522 helpline calls by regions (per 1000 people, 2013–2020)

In the regional analysis, the unit of interest is not the day anymore but the region-year combination. Furthermore, the temporal coverage is wider (2013–2020), and we can leverage cross-regional variation in potential socioeconomic confounders which vary greatly by year (while less so by day), such as GDP per capita and unemployment rate. Figure 3 visualizes the population-adjusted number of 1522 calls for the combined period of March-June and for years 2013 (earliest, left) and 2020 (latest, right). While the highest call rate is between 0.20 and 0.25 per 1000 people in 2013, it climbs up to 0.30–0.35 per 1000 in 2020 in the post-lockdown period. In particular, we observe a notable increase in helpline call rates in those regions that were severely impacted by the first wave of the pandemic such as Lombariay, Piemonte, Lazio, and Emilia-Romagna. Having data for each region, we conducted a first preliminary investigation to explore whether the predictive power of Google searches for the keyword *1522* differs by region. Excluding one region at a time to preserve sample size, we found that there is little cross-regional heterogeneity in the predictive power of Google searches (Figure A2 in the Online Appendix).

**Fig. 4:**
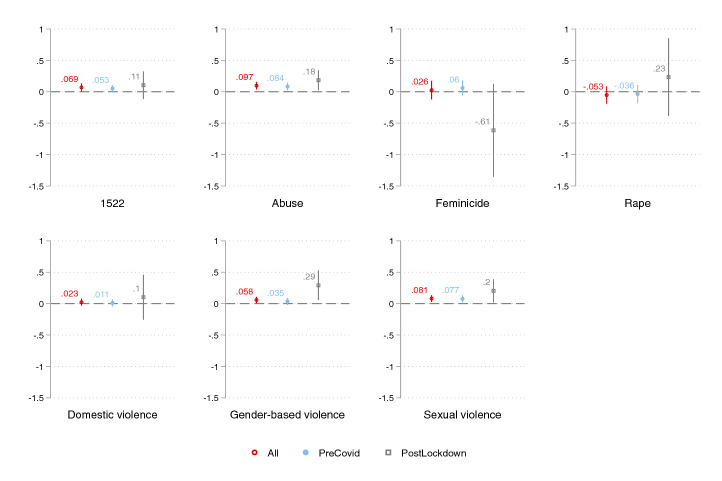
Coefficient plot from regressions of yearly 1522 calls on Google searches, by selected keywords (regional-level)

Figure 4 presents results obtained from regional-level data on yearly-aggregated number of calls by victims and users combined.^22^ Victims are defined by ISTAT as “people who called 1522 to ask for help for themselves and suffered violence in one of its various forms,” while users are defined as “people who called 1522 to ask help for themselves or others.” Search frequencies for keywords *1522*, *abuse*, *gender-based violence* and *sexual violence* are positively and significantly associated with the number of anti-violence helpline calls, while results for *domestic violence* suggest positive yet non-significant associations. Conversely, searches for keywords *feminicide* and *rape* appear to be insignificant and negatively associated with the number of 1522 calls (full results on the whole sample in the Online Appendix Table A9). Relying on additional information on whether the call made is a first versus subsequent call, we also find that the predictive power of Google searches is significantly more relevant for first calls (Table A10), underscoring once again the informational channel we outlined in the introduction. This suggests that Google is really a way for threatened women to get access to first-hand information. Once they hold this information, they likely do not rely on Internet anymore but turn to more “traditional” and effective sources of help. In this same table we show results separately for whether the person calling is a victim or a user. This additional panel does not highlight marked differences in the significance of the estimates, yet the magnitude of the coefficients is higher among users, perhaps suggesting that (i) some victims actually identify as users due to stigma, and/or (ii) Google is a more useful device for individuals who face potential—rather than actual threats. Models on the pooled sample with a year=2020 dummy (our proxy for post-lockdown in the regional yearly analyses) interacted with each keyword (Table A11) show that for the same set of keywords outlined above, namely *1522*, *abuse*, *gender-based violence* and *sexual violence*, the association between searches and calls is significantly stronger in the post-lockdown period in terms of both magnitude and statistical significance. Together, these findings signal the particular relevance of Google search engines for women seeking help during confinement periods in which traditional help mechanisms become harder to reach.

### Daily Regional Emergency Calls in Lombardy

**Fig. 5:**
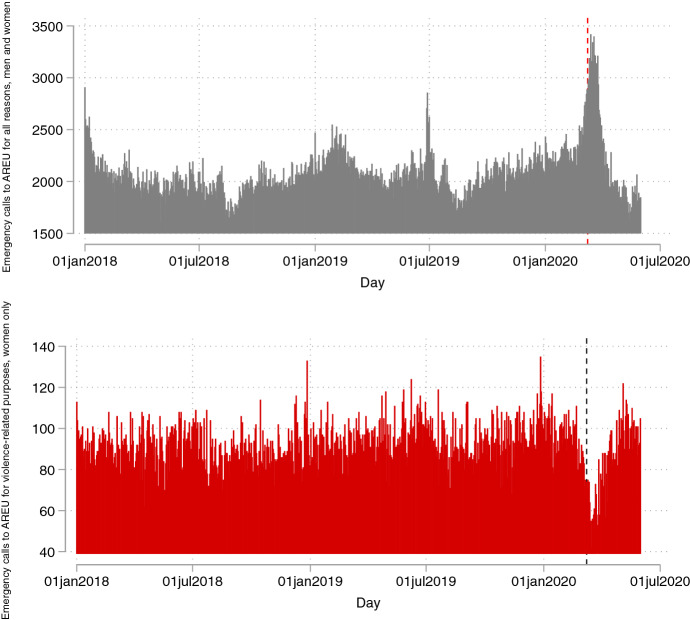
Daily number of calls to AREU from men and women for all reasons combined (top panel) and from women for accident or violence-related purposes (bottom panel)

Figure 5 reports the daily number of calls to AREU—calls made to the Italy-wide emergency number in Lombardy to request an ambulance—from men and women for all reasons combined (top panel) and from women for accident or violence-related purposes (bottom panel). Trends are very different across the two panels, especially around the lockdown period. The bottom panel shows a marked drop in calls to AREU made by women for accident or violence-related purposes in the immediate post-lockdown period—a piece of evidence which stands in contrast with trends for 1522 calls shown in Fig. 1 and discussed in Colagrossi et al. (2020), and with trends in calls to AREU made by both men and women for all reasons combined (Fig. 5, top panel). One hypothesis is that in the wake of strict confinement measures, the threat of violence might increase importantly, hence women resort to the main IPV helpline (1522) to seek help and information, rather than requesting an ambulance (AREU), which would rather occur in the presence of actual violence. Also, given the general state of emergency, it is likely that lines for the emergency number were congested for other reasons (this clearly emerges from Fig. 5, top panel), hence most calls could not go through and IPV victims resorted to the primary helpline (1522) irrespective of type of threat. Alternatively, it may be the case that women calling do not provide the exact reason underlying the emergency (e.g., due to stigma), thus leading to mis-recording and/or mis-reporting of information on reasons behind the calls.

**Fig. 6:**
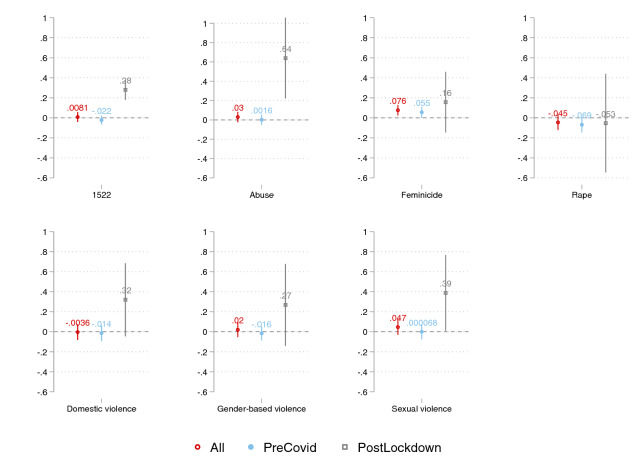
Coefficient plot from regressions of daily calls to AREU on Google searches, by selected keywords (Lombardy), 1-week lag

We last test the relationship between online searches and daily calls to the emergency number in Lombardy using daily data from AREU. Note, once again, that emergency calls to AREU differ from 1522 calls as the former are aimed at requesting an ambulance, thus measuring actual violent cases that require immediate help and assistance. Consistently with such discrepancy, our results with AREU data—reported in Fig. 6—are rather different from the above. While the sign of the estimated coefficients is for the most part positive and aligned with the above, only searches for the keyword *feminicide* positively and significantly predict emergency calls to AREU for the whole period considered (empty dot)—full results in the Online Appendix Table A12. Estimates are also robust to including month fixed effects (Table A13) and clustered standard errors at the month level (Table A14). However, the evidence changes drastically when restricting the focus to the post-lockdown period (empty square). The estimated coefficient on online searches gets two to six times bigger in magnitude for all keywords except for *rape*, and the coefficient becomes statistically significant for the keywords *1522, abuse, domestic violence* and *sexual violence*. Note that three of the four keywords—namely *1522*, *abuse*, and *sexual violence*—are the same ones that become more strongly significant when predicting 1522 calls in the post-lockdown period in Fig. 4—full results reported in Table A15. These findings imply that the tendency to seek IPV-related help online and reported IPV emergencies are more aligned during the lockdown period as Google, and Internet in general, become major sources for information-seeking during confinement periods. Overall, our core results combined seem to suggest that online searches are a powerful tool to track potential threats of IPV before and after global-level crises such as the current COVID-19 pandemic—with stronger predictive power post-crisis—while online searches help predict actual violence *only* in post-crises scenarios.

## Validation of Keywords

Despite their common appearance in existing scholarship and popular presence in the international IPV discourse, we acknowledge that the keywords chosen might not fully represent the type of keywords that women would look for if faced with a threat of IPV, or might just be representative of searches conducted by specific population strata (e.g., high-SES women). We thus decided to validate our keywords by asking people directly which keywords they would look for if they were to search for help and/or information on Google. We achieved this goal by running a short online survey targeting the population 18+ in Italy through a computer-assisted web interviewing (CAWI) procedure and the Facebook Application Programming Interface (API).^23^ The survey—completely anonymous—lasted approximately five minutes and included two open-ended questions in which respondents could freely write the first three words they would search for in Google if they were to seek information or help on IPV,^24^ and a battery of 19 pre-selected keywords from which respondents could select a maximum of 3. This battery included the nine keywords that we selected from the review of the existing literature—and feature in the core analyses—plus 10 other keywords extracted from Italian newspapers, newscasts, and qualitative interviews carried out with experts (e.g., members of the executive committee of AREU Lombardy, and volunteers from anti-violence centers both interviewed in January 2021) and researchers who have previously dealt with issues of IPV in Italy (interviewed in December 2020 and January 2021). The rationale behind the choice to include 10 additional keywords on top of the nine selected *a priori* was twofold. On one hand, we wanted to verify that the words selected ex-ante were also suitable to our study setting and produced enough digital “results" to serve as valid predictors. On the other hand, we wanted to be sure that we were not missing anything specific to the Italian context such as, for instance, words that pertain to colloquial jargon (though excluding dialectal words and expressions).

We looked both at the frequency of words reported by the Facebook survey respondents in the two open-ended questions (Figure A3) and at the words selected among the battery of 19 items proposed (Figure A4). Reassuringly, some words that appear most frequently in the open-ended questions such as *domestic violence* (violenza domestica) and *feminicide* (femminicidio) were already included among the ex-ante keywords employed in the core empirical analyses, thus validating some of our initial choices. Conversely, three frequently cited words that were not included in the ex-ante set of keywords were *anti-violence center* (centro antiviolenza), *woman* (donna) and *help* (aiuto). In the analyses that follow we focus on the former as a unique keyword, while the latter two are combined in no specific order (*woman & help*) given that, if taken individually, they would not be exclusively IPV-related.

When looking at the frequency of the words chosen among the list of 19 keywords proposed, we find that 55% of respondents selected *domestic violence* as the most common keyword—one of the keywords that features most prominently in our core analyses and in the word cloud (Figure A3)—followed by *violence & home* (37%), *violence & help* (32%), *violent partner* (29%), *violence & rape* (28%), *abuse* (16%), and *violence* (15%). While *abuse* was also already included in the keywords selected ex-ante, the keywords including “violence” were not. We therefore downloaded them, together with *anti-violence center* and *woman & help* from the open-ended questions, for the same period under investigation. For some keywords, the Google Trends search query provided either partial results for more recent times or no results at all due to low search volumes. We thus only kept the three violence-related keywords with a satisfactory search volume, namely *violent partner*, *violence & help*, and *violence & home*.^25^

We then replicated the national-level analyses with our complete dataset by using the daily number of valid 1522 calls and daily number of Google hits for those keywords that were not included ex-ante but emerged as popular choices subject to adequate search volume on Google Trends. Results from this exercise are reported in Figure A5 in the Appendix, which shows that the two keywords that are positively and significantly correlated with 1522 calls are *anti-violence center* and *violence & home* which are, in fact, the two keywords exhibiting the highest correlations with Google searches for “1522” (Table A17)—the keyword with the strongest and most consistent predictive power across the three data sources in the core analyses. Overall, this validation exercise demonstrated that our initial choice of keywords was rather adequate and performed quite well, especially for some very popular keywords such as *domestic violence*, *abuse*, and *feminicide*, yet we neglected a couple of important dimensions that individuals actively search for, namely *anti-violence center* (emerging from the open-ended questions summarized in the word cloud) and *violence & home*. Interestingly, more colloquial expressions such as *hit* (menare) and *blows* (botte) did not emerge as popular choices.

### Digital Divides

While the predictive potential of digital traces left by users on search engines is often overlooked, digital traces might be less suited to understanding the existence and magnitude of digital divides. We conclude this investigation by exploring whether the predictive potential of digital traces differs for particular groups of users. The existence of *third-level* digital divides suggests that the ability to leverage the potential of specific digital technologies (e.g., search for the correct keyword or interpret and leverage the information received in the best possible way) may vary widely by socioeconomic status. It is therefore crucial to know, or at least infer, for which categories of people digital traces may be more (or less) effective towards a specific goal (Olteanu et al., 2019).

The best way to overcome this issue would be to leverage advanced artificial intelligence algorithms that take into account the different online behaviors of different categories of the population (Dargin et al., 2021). As promising as this might sound, its scalability in terms of applied social research is challenged by the complexities inherent in such models. A simpler way is to combine the analysis of digital traces with survey data in order to understand *who* uses the technology to obtain *what* type of information—the strategy we follow here. Specifically, the data obtained from the Facebook survey allow us to explore if specific characteristics of the respondents, such as the level of education, are associated with the likelihood of searching one keyword versus another.

**Fig. 7:**
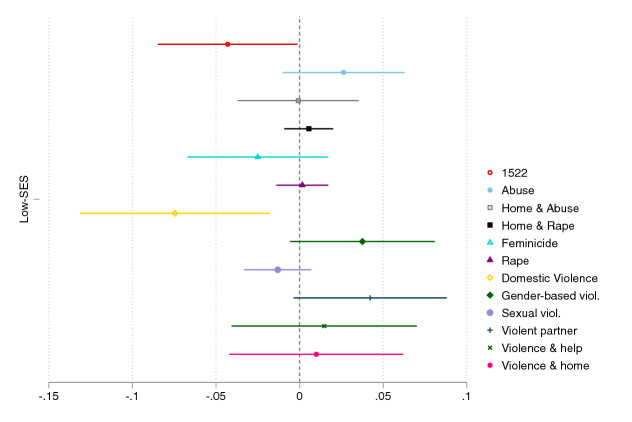
Coefficient plot from regressions of Google searches on a dummy for low-SES (high-school diploma or less). Facebook survey

Figure 7 plots coefficients from a simple OLS regression of Google searches—the nine keywords included in the core analyses plus the three identified as popular from the closed-ended question—on a dummy taking value 1 if the female respondent has completed high-school diploma or less (an appropriate threshold for “low-SES” in the Italian context), while controlling for age and current marital status and using appropriate post-stratification weights. The figure provides evidence of a negative association between low-SES and searches for keywords *1522*, *domestic violence*, *feminicide*, and *sexual violence*, with statistically significant coefficients for the former two keywords. This finding suggests that there are keywords—and primarily those that exhibit high predictive power in the core analyses across the three data sources—which are more likely searched by high-SES individuals. Most other keywords are positively associated with the low-SES dummy, yet coefficients are not statistically significant. Examples of keywords with low predictive power that are positively associated with the low-SES dummy are *home & abuse*, *home & rape*, *violent partner*, and *violence & help*. One exception to this SES-cleavage whereby higher-SES individuals search for keywords that are more “consequential” is *gender-based violence*, which emerges as rather relevant from the core analyses, yet it exhibits a positive association with low-SES. Overall, results from this simple exercise suggest that high-SES individuals are, as hypothesized (*HP3*), more likely to use search engines more effectively than their low-SES peers, corroborating the existence of *third-level* digital divides.

## Conclusions and Discussion

This study addressed the question of whether big data might help to reach “hard-to-reach” populations such as victims of intimate partner violence using Italy as a case study. We explored three related research questions. First, we investigated whether online searches help predict instances of IPV in Italy over the last half decade. Second, we assessed whether the predictive power of online searches is higher, lower, or unaltered in the wake of global-level crises such as the current COVID-19 pandemic. To do so, we relied on search frequencies for multiple keywords measuring different facets of IPV, and we combined three different data sources varying in terms of temporal coverage and level of analysis. By combining multiple sources of data, we were also able to characterize instances of IPV into *potential* violence (or “threat” of violence) and *actual* violent cases as measured by calls to request an ambulance. Lastly, we explored whether there are socioeconomic differences in terms of who benefits the most from being online and from online search behavior related to IPV. To this end—and in order to validate our *a priori* selection of nine keywords that we deemed relevant in an international comparative context—we designed a new Facebook survey asking respondents directly which keyword they would search for in Google if they were to look for information and/or help on IPV.

Starting from the first research question, our findings at the country-level suggest that online searches using selected keywords well predict daily calls to domestic-violence helplines. The same overall finding is confirmed by regional-level analyses predicting yearly—rather than daily—calls, even after controlling for regional-level controls such as GDP per capita, unemployment rate, and educational attainment. Conversely, analyses on daily calls to the emergency number in Lombardy—proxying for actual threats of violence—provide little evidence of predictive power of online searches, except for the keyword *feminicide* which, reasonably, turns out to be the most closely related to an ambulance request. These findings combined suggest that digital traces emerge as a powerful tool to track the risk of potential violence in “normal” or non-crises circumstances, while they are seemingly less effective at tracking actual violent cases reported.

Moving to the second research question, while country-level analyses show little evidence of differential associations in the post-lockdown period, regional- and Lombardy-level analyses do suggest far stronger associations—both in terms of magnitude and statistical significance—in the post-lockdown period, with a high degree of concordance in terms of the most relevant keywords. Two are the implications of these findings. First, these results underscore the key relevance of search engines—and of online connectivity in general—for women seeking help during confinement periods in which traditional help mechanisms become harder to reach. Second, findings from Lombardy which showed little to no associations in non-crises times (i.e., pre-lockdown) in fact reveal that online searches can also be a powerful tool to track *actual* violent cases in situations of global crises such as the current COVID-19 pandemic. As such, we are hopeful that policymakers will take these findings at face value and rely more on these types of data and analyses to think about how to best devise surveillance/monitoring systems to contain, minimize, and even anticipate surges in IPV, as well as reflect on how to allocate financial resources targeted towards the management of IPV.

Lastly, our novel short Facebook survey provided an opportunity to validate our ex-ante choice of keywords, suggesting that we had properly identified at least some of the most popular keywords that individuals in Italy would search for in Google. Most importantly, additional analyses targeted specifically towards identifying SES-differences in online behavior suggested that the keywords with lower predictive power were overwhelmingly chosen by low-SES individuals, while high-SES more frequently selected the keywords with strongest predictive power. This is an important and novel finding, which suggests that high-SES individuals are, as hypothesized, more likely to use search engines more effectively than their low-SES counterparts. On a broader level, a finding of this kind corroborates the existence of *third-level* digital divides in Italy, supporting the idea that some individuals are indeed better equipped to leverage the full potential of specific digital technologies towards a specific end—in this case, looking for IPV-related information or seeking help through IPV helplines and/or emergency numbers.

To conclude, results from this study suggest that Google searches using selected keywords measuring different aspects of IPV may serve as powerful tools for tracking *potential* threats of IPV before and after global-level crises such as the COVID-19 pandemic—with stronger predictive power post-crisis—while online searches help predict *actual* violence in post-crises scenarios only, likely pointing towards a more active use of the Internet and the online resources that the Internet offers. As a matter of fact, while actual reporting of IPV tends to be lower in times of crises, online connectivity tends to be high, either due to rising unemployment, forced lockdowns at home, or both. We thus conclude that big data might be a very important—and, to date, widely underappreciated—resource for tracking or even anticipating IPV and getting a real-time picture of the brunt of domestic violence that women bear every day, but especially so in the wake of global-level crises. There is an important caveat, though, as our evidence also suggests that effective forecasting may be far more reliable among high-SES population strata. It is therefore crucial to devise policies aimed at closing all types of digital divides, not just those related to access (*first-level*) or knowledge (*second-level*).

## Supplementary Information

Below is the link to the electronic supplementary material.

Supplementary file 1 (pdf 1053 KB)

## Footnotes

1 This feature is currently available for searches made within the U.S. https://blog.google/products/search/connecting-people-domestic-violence-support/.

2 Although comprehensive data on IPV have been advocated for several years from different sources, it is still difficult to find internationally comparable data. One of the reasons behind this difficulty is that IPV is a largely culturally-driven phenomenon. The most recent comparative survey (https://fra.europa.eu/en/publication/2014/violence-against-women-eu-wide-survey-main-results-report) dates back to 2014. According to these data, in the 28 EU countries gender abuse is more widespread in Northern Europe. Denmark lies at the top of the ranking, where 52% of the women interviewed said they had suffered physical or sexual violence since 15. In this ranking, Italy takes the eighteenth position (27%).

3 van Deursen and Helsper (2015) define third-level digital divides as “disparities in the returns from Internet use within populations of users who exhibit broadly similar usage profiles”.

4 https://www.istat.it/it/violenza-sulle-donne/speciale-covid-19.

5 https://www.corriere.it/cronache/20_aprile_13/coronavirus-donne-vittime-violenza-ora-chiedono-aiuto-via-mail-30b8152a-7c97-11ea-9e96-ac81f1df708a.shtml.

6 https://www.nytimes.com/2020/04/06/world/coronavirus-domestic-violence.html.

7 https://edition.cnn.com/2020/03/27/health/domestic-violence-coronavirus-wellness-trnd/index.html.

8 http://www.governo.it/it/media/campagna-di-comunicazione-libera-puoi/14459.

9 The Libera Puoi campaign, promoted by the Department for Equal Opportunities in support of women victims of violence during the Coronavirus emergency, was intended to launch a message of closeness to remind women isolated at home that in case of difficulty they would not be alone and could count on the free anti-violence and anti-stalking number 1522, on shelter houses, and on anti-violence centers.

10 https://gs.statcounter.com/search-engine-market-share/all/italy.

11 https://www.agcom.it/documents/10179/20440899/Documento+generico+16-10-2020/dcdbcb3a-720c-4878-9f10-dcd8912ba984?version=1.0.

12 In this study, we define IPV as violence perpetrated against women by their male partners within and outside of marriage. We do acknowledge that IPV is not limited to women and/or can be occurring within same-sex partnerships, yet we adopt this definition in this study for two reasons. First, recent data from helpline calls from Italy suggest that victims, 40% of callers, were women in 97% of cases and this has been a constant figure over time. Therefore, we only kept calls to helplines made by women as main outcomes of our analyses. Second, Google Trends deliver no results when looking for keywords that more closely relate to violence against men, thus providing no reliable predictors.

13 As always when working with IPV-related data, we acknowledge that the outcomes might well suffer from under-reporting.

14 These combinations refer to search queries that include both words, in no particular order.

15 We will use the English label in all figures and tables that follow.

16 Some keywords are missing in some regions due to the low volume and frequency of Google searches that fail to pass the threshold set by Google Trends algorithm and thus reported as zero. See Table A1 for detailed information on missing keywords by regions.

17 Available at: https://www.istat.it/it/archivio/246557.

18 Albeit not perfect, we imposed the following restrictions to identify violence-related calls: (i) we kept only “home” as the location from which the call was made; (ii) we kept only women as the sex of the caller; (iii) we restricted the age range to 10–85; (iv) we only kept violence-related motives (e.g., we excluded respiratory motives, among others).

19 For conciseness, we run all these robustness checks only for Italy-wide analyses using data from the Equal Opportunity Department, yet robustness checks conducted on the other two sources of data—ISTAT and AREU—deliver essentially analogous results.

20 Due to the lack of adequate number of observations, inquiries for *home & abuse* and *home & rape* are excluded from regional analysis.

21 Such as search for legal information, out-of-target calls related to requests for other useful phone numbers, information about national shelters for victims of violence, and other reasons.

22 Analyses could only be conducted for seven out of the nine keywords, as too few queries could be produced for the keywords *home & abuse* and *home & rape* for the selected geographical unit and time frame.

23 A description of the procedures implemented during the survey is provided in the Online Appendix. Note that the sample was made nationally representative of the Italian population in terms of demographics using pot-stratification weights, as commonly done with online surveys. See, for instance, Alexander et al. (2022) and Battiston et al. (2021).

24 (1) “Imagine you are seeking information on Google about intimate partner violence. Which keywords would you use and insert in the search engine?” and (2) “Imagine you are seeking help and support on Google about intimate partner violence. Which keywords would you use and insert in the search engine (Please, list the three main words that come to your mind)?”.

25 For the daily data downloaded at the country level, *violence & rape* and *violent partner* (female) did not produce any result. *Violent partner* (male) and *violence & help* had missing daily data in earlier years but complete weekly data.
