# Supplementary material for: Harnessing the Potential of Google Searches for Understanding Dynamics of Intimate Partner Violence Before and After the COVID-19 Outbreak
Source: Eur J Popul. 2022 May 30;38(3):517–45. doi: 10.1007/s10680-022-09619-2 (PMC9150629; doi:10.1007/s10680-022-09619-2)
Supplement: Supplementary file 1 — (pdf 1053 KB) [file 10680_2022_9619_MOESM1_ESM.pdf]

Online Appendix

1 The first wave of the COVID-19 outbreak in Italy

On Friday February 21st, 2020, the first case of COVID-19 was diagnosed in a man living in the town of Codogno in the province of Lodi, a city located in the Northern region of Lombardy in Italy. The virus then spread across neighboring regions in Northern Italy – including Veneto, Emilia Romagna, and Piedmont, all of which began to report rapid increases in cases. Two days later, on February 23, the government issued a decree which prohibited the movement of people outside 10 municipalities located in Lombardy and a municipality in Veneto. From March 8, restrictions to avoid any movement were extended to the whole of Lombardy and to other fourteen provinces in Northern Italy. On March 10 — our cutoff point for the definition of lockdown in the current study — a new decree issued by the Prime Minister extended these lockdown measures across the entire country of Italy until May 4, when the country started a mild reopening which was completed by mid-June 2020. The spread of the virus across the country – at least for the first wave of the COVID-19 pandemic — was uneven with the majority of cases (and deaths) being concentrated in Lombardy — a region with a population of approximately 10 million people — which, as of January 2021, counts more than 30% excess deaths (hence our focus on Lombardy in the latter part of the analysis). At the beginning of January 2021, Italy is the eighth country in the world and the fourth in Europe for total number of COVID-19 cases, and the fifth country in the world and the first in Europe for total number of COVID-related deaths.

2 Additional details on data and methods

Google Trends data: As Google Trends only allow for daily data to be obtained for a period of three months (90 days), we repeatedly obtained daily Google Trends data for each three-month period to achieve the time frame required. Once the consecutive periods of three months were pieced together, we addressed the issue of each part of data being normalized within itself. Thus, we downloaded weekly data for the given time frames (at once, to ensure that the normalization is within the necessary time frame) and matched the weekly and daily data where a value was present for the same date in both data sets. Based on the matching dates, we calculated a weekly adjustment factor (Risteski & Davcev, 2014). By multiplying the daily data by the relevant weekly adjustment factor, we ensured the consistency

of the data set of daily data, and ultimately normalized it on a 0-100 range.

Regional-level analysis with controls at the regional level: As of December 2020, educational attainment and annual unemployment data at the regional level are available until the year 2019. Therefore, we replaced data on these two controls for 2020 with data from 2019 as closest proxy. Moreover, we replaced regional GDP per capita of the years 2019 and 2020 with the one of 2018 since the data are present only up to 2018.

Table A1: Missing keywords by regions

Keywords	*N of missing*	*Missing regions*
1522	2	Molise,Valle D'Aosta
Abuse	1	Valle D'Aosta
Feminicide	1	Valle D'Aosta
Rape	-	-
Domestic violence	7	Abruzzo, Basilicata, Friuli, Molise, Trento, Umbria, Valle D'Aosta
Gender-based violence	3	Basilicata, Trento Alto Adige, Valle D'Aosta
Sexual violence	3	Molise,Valle D'Aosta, Umbria

3 Results using daily data from the Equal Opportunity Department (Presidency of the Italian Council of Ministers), Italy

Table A2: Google hits and number of valid calls: Daily data, Italy (1-week lag)

	(1)	(2)	(3)	(4)	(5)	(6)	(7)	(8)	(9)
					Valid 1522 calls				
1522	0.590***								
	(0.186)								
Abuse		0.058							
		(0.108)							
Home & Abuse			-0.039						
			(0.062)						
Home & Rape				0.001					
				(0.061)					
Feminicide					0.397***				
					(0.086)				
Rape						-0.003			
						(0.068)			
Domestic violence							0.408***		
							(0.078)		
Gender-based violence								0.234***	
								(0.077)	
Sexual violence									0.040
									(0.061)
Year FE	✓	✓	✓	✓	✓	✓	✓	✓	✓
Observations	601	601	601	601	601	601	601	601	601

Note: OLS. Robust standard errors reported in parentheses. The models follow Eq. 1 in the methods section. * $p<0.10$, ** $p<0.05$, *** $p<0.01$

Table A3: Google hits and number of valid calls: Daily data, Italy, with year and month fixed effects (1-week lag)

	(1)	(2)	(3)	(4)	(5)	(6)	(7)	(8)	(9)
	Valid 1522 calls								
1522	0.562***								
	(0.169)								
Abuse		0.041							
		(0.112)							
Home & Abuse			-0.037						
			(0.060)						
Home & Rape				-0.018					
				(0.061)					
Feminicide					0.472***				
					(0.099)				
Rape						-0.012			
						(0.074)			
Domestic violence							0.379***		
							(0.073)		
Gender-based violence								0.260***	
								(0.078)	
Sexual violence									0.034
									(0.065)
Year FE	✓	✓	✓	✓	✓	✓	✓	✓	✓
Month FE	✓	✓	✓	✓	✓	✓	✓	✓	✓
Observations	601	601	601	601	601	601	601	601	601

Note: OLS. Robust standard errors reported in parentheses. The models follow Eq. 1 in the methods section. * p<0.10, ** p<0.05, *** p<0.01

Figure A1: Coefficient plot from regressions of daily 1522 valid calls on Google searches, by selected keywords (whole Italy), contemporaneous predictors.

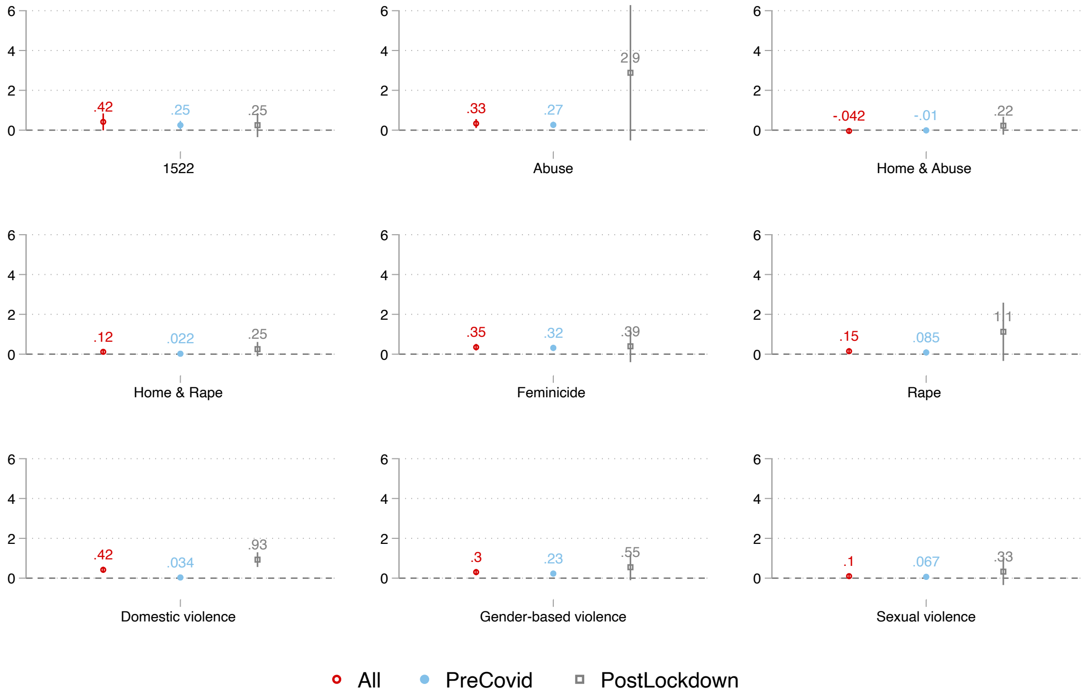

Notes: Data on the outcome from the Equal Opportunity Department, Presidency of Italian Council collected daily from March 1, to June 30, from 2016 to 2020. Predictors are measured at the same time as the outcome (contemporaneous). 95% confidence intervals. The models follow Eq. 1 in the methods section.

Table A4: Google hits and number of valid calls: Daily data, Italy, with clustering of standard errors (SE) at the month level (1-week lag)

	(1)	(2)	(3)	(4)	(5)	(6)	(7)	(8)	(9)
	Valid 1522 calls								
1522	0.590***								
	(0.186)								
Abuse		0.058							
		(0.108)							
Home & Abuse			-0.039						
			(0.062)						
Home & Rape				0.001					
				(0.061)					
Feminicide					0.397***				
					(0.086)				
Rape						-0.003			
						(0.068)			
Domestic violence							0.408***		
							(0.078)		
Gender-based violence								0.234***	
								(0.077)	
Sexual violence									0.040
									(0.061)
Year FE	✓	✓	✓	✓	✓	✓	✓	✓	✓
Observations	601	601	601	601	601	601	601	601	601

Note: OLS. SE clustered at the month level reported in parentheses. The models follow Eq. 1 in the methods section. * p<0.10, ** p<0.05, *** p<0.01

Table A5: Google hits and number of valid calls: Daily data, Italy, with post-lockdown interaction (1-week lag)

	(1)	(2)	(3)	(4)	(5)	(6)	(7)	(8)	(9)
					Valid 1522 calls				
1522	0.313***								
	(0.101)								
Post lockdown=1 × 1522	0.301								
	(0.308)								
Abuse		0.074							
		(0.104)							
Post lockdown=1 × Abuse		-0.374							
		(1.933)							
Home & Abuse			0.005						
			(0.034)						
Post lockdown=1 × Home & Abuse			-0.307						
			(0.258)						
Home & Rape				-0.023					
				(0.040)					
Post lockdown=1 × Home & Rape				0.287					
				(0.231)					
Feminicide					0.269***				
					(0.062)				
Post lockdown=1 × Feminicide					0.930**				
					(0.471)				
Rape						-0.020			
						(0.051)			
Post lockdown=1 × Rape						1.163			
						(0.734)			
Sexual violence							0.036		
							(0.046)		
Post lockdown=1 × Sexual violence							0.574		
							(0.369)		
Gender-based violence								0.081	
								(0.050)	
Post lockdown=1 × Gender-based violence								0.535*	
								(0.323)	
Domestic violence									0.042
									(0.045)
Post lockdown=1 × Domestic violence									0.977***
									(0.203)
Post lockdown=1	80.027***	87.180***	88.524***	83.370***	80.173***	76.172***	76.281***	76.027***	63.322***
	(6.201)	(10.782)	(5.614)	(5.603)	(5.949)	(8.190)	(8.102)	(7.976)	(7.079)
Year FE	✓	✓	✓	✓	✓	✓	✓	✓	✓
Observations	601	601	601	601	601	601	601	601	601

Note: OLS. Robust standard errors reported in parentheses. The models follow Eq. 1 in the methods section, yet with an additional dummy *post* lockdown, alongside *post**keyword interactions. * p<0.10, ** p<0.05, *** p<0.01

Table A6: Google hits and number of valid calls: Daily data, Italy, Poisson regression (1-week lag)

	(1)	(2)	(3)	(4)	(5)	(6)	(7)	(8)	(9)
					Valid 1522 calls				
1522	0.005***								
	(0.002)								
Abuse		0.001							
		(0.002)							
Home & Abuse			-0.001						
			(0.001)						
Home & Rape				0.000					
				(0.001)					
Feminicide					0.006***				
					(0.001)				
Rape						-0.000			
						(0.001)			
Domestic violence							0.005***		
							(0.001)		
Gender-based violence								0.003***	
								(0.001)	
Sexual violence									0.001
									(0.001)
Year FE	✓	✓	✓	✓	✓	✓	✓	✓	✓
Observations	601	601	601	601	601	601	601	601	601

Note: Poisson Regression. Robust standard errors reported in parentheses. The models follow Eq. 1 in the methods section, yet estimated through a Poisson model. * p<0.10, ** p<0.05, *** p<0.01

Table A7: Google hits and number of valid calls: Daily data, Italy, with dummy for Libera Puoi awareness campaign (1-week lag)

	(1)	(2)	(3)	(4)	(5)	(6)	(7)	(8)	(9)
	Valid 1522 calls								
1522	0.604***								
	(0.182)								
Abuse		0.031							
		(0.098)							
Home & Abuse			-0.050						
			(0.057)						
Home & Rape				0.008					
				(0.056)					
Feminicide					0.403***				
					(0.091)				
Rape						0.040			
						(0.064)			
Domestic violence							0.343***		
							(0.072)		
Gender-based violence								0.159**	
								(0.065)	
Sexual violence									0.081
									(0.055)
Post lockdown	67.059***	73.564***	73.794***	73.611***	70.987***	73.652***	71.767***	72.862***	74.159***
	(10.112)	(9.930)	(9.860)	(9.980)	(9.838)	(9.903)	(9.082)	(9.979)	(9.925)
Libera Puoi	22.651**	17.852*	17.806*	17.846*	19.877**	17.897*	16.339*	17.440*	17.787*
	(10.006)	(10.139)	(10.087)	(10.170)	(9.999)	(10.117)	(9.512)	(10.067)	(10.105)
Year FE	✓	✓	✓	✓	✓	✓	✓	✓	✓
Observations	601	601	601	601	601	601	601	601	601

Note: OLS. Robust standard errors reported in parentheses. The models follow Eq. 1 in the methods section, yet with an additional dummy *post* lockdown and an additional dummy for the *Libera Puoi* campaign. * p<0.10, ** p<0.05, *** p<0.01

Table A8: Google hits and number of valid calls: Daily data, Italy, Placebo (1-week lag) test

	(1)	(2)
	Valid 1522 calls	
Pizza home delivery	0.112	
	(0.080)	
Zumba		0.107
		(0.074)
Year FE	✓	✓
Observations	601	601

Note: OLS. Robust standard errors reported in parentheses. The models follow Eq. 1 in the methods section. * p<0.10, ** p<0.05, *** p<0.01

4 Results using yearly data from ISTAT, regional-level

Figure A2: Coefficient plot from regressions of yearly 1522 calls on Google searches for keyword 1522, excluding one region at a time.

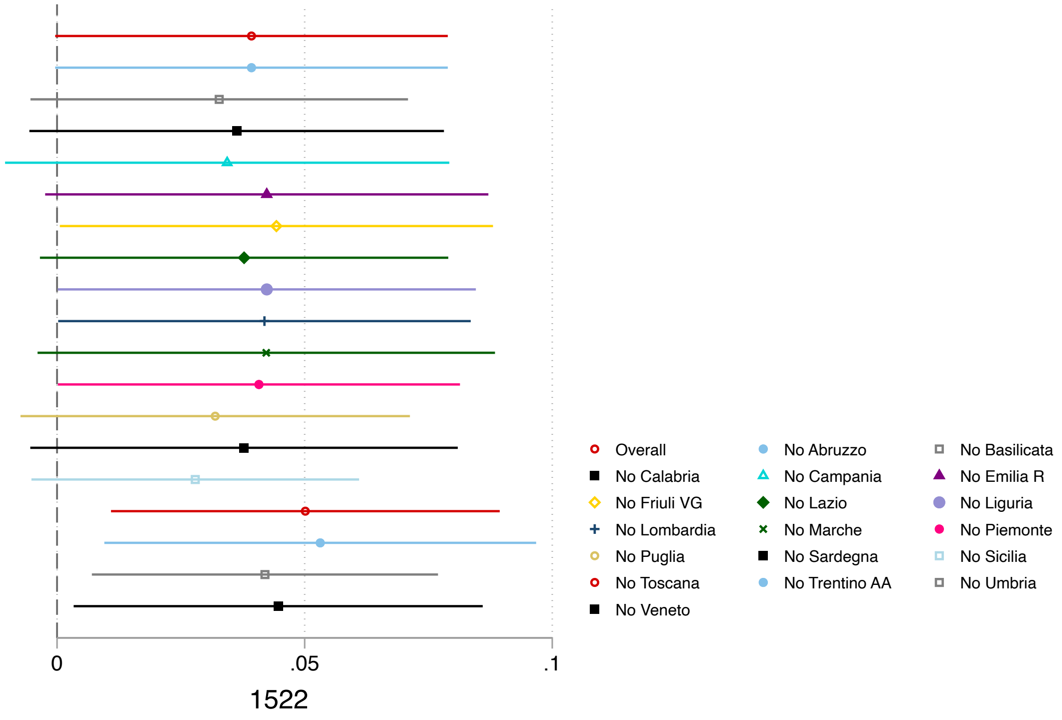

Notes: Data on the outcome from ISTAT collected for the period March-June 2013-2020 (one observation per year) and aggregated at the regional level. 95% confidence intervals. Estimated coefficients have been multiplied by 100 to obtain a magnitude that is comparable to the one observed across the other two data sources. The models follow the specification provided in Eq. 2, yet excluding one region at a time to preserve sample size. Regions Valle d'Aosta and Molise do not appear in this graph, as no queries were obtained for keyword *1522* in these two regions (hence the estimated coefficient would be identical to the overall one).

Table A9: Google hits and 4 months-aggregated calls at the regional level

	(1)	(2)	(3)	(4)	(5)	(6)	(7)
	Valid 1522 calls						
1522	0.069**						
	(0.031)						
Abuse		0.097***					
		(0.028)					
Feminicide			0.026				
			(0.071)				
Rape				-0.053			
				(0.067)			
Domestic violence					0.023		
					(0.025)		
Gender-based violence						0.058**	
						(0.024)	
Sexual violence							0.081***
							(0.025)
Regional controls	✓	✓	✓	✓	✓	✓	✓
Year FE	✓	✓	✓	✓	✓	✓	✓
Observations	144	152	152	160	104	136	136

Note: OLS, as indicated. Clustered standard errors at the regional level reported in parentheses. The models follow Eq. 2 in the methods section. The number of observations varies by model as the availability of yearly queries varies by keyword. Estimated coefficients have been multiplied by 100 to obtain a magnitude that is comparable to the one observed across the other two data sources. * p<0.10, ** p<0.05, *** p<0.01

Table A10: Heterogeneous results: Google hits and 4 months-aggregated calls at the regional level by frequency of call and type of user

	Frequency of call		*Type of caller*	
	First time	Other	Victims	Users
1522	0.045**	0.014	0.022**	0.048**
	(0.015)	(0.008)	(0.01)	(0.021)
Abuse	0.052***	0.040***	0.026***	0.071***
	(0.020)	(0.009)	(0.008)	(0.021)
Feminicide	-0.016	0.004	0.011	0.015
	(0.041)	(0.014)	(0.029)	(0.042)
Rape	-0.004	-0.005	-0.019	-0.034
	(0.041)	(0.014)	(0.028)	(0.039)
Domestic violence	0.029	0.007	0.010	0.013
	(0.023)	(0.008)	(0.009)	(0.017)
Gender-based violence	0.042**	0.015	0.019**	0.039**
	(0.018)	(0.009)	(0.007)	(0.018)
Sexual violence	0.047**	0.025***	0.024**	0.057***
	(0.020)	(0.008)	(0.009)	(0.017)
Regional controls	✓	✓	✓	✓
Year FE	✓	✓	✓	✓

Note: OLS, as indicated. Each cell represents a distinct regression and sample sizes vary (omitted, as they are reported in above tables). Clustered standard errors at the regional level reported in parentheses. The models follow Eq. 2 in the methods section. The number of observations varies by model as the availability of yearly queries varies by keyword; please refer to Table A9 for number of observations. Estimated coefficients have been multiplied by 100 to obtain a magnitude that is comparable to the one observed across the other two data sources. * p<0.10, ** p<0.05, *** p<0.01

Table A11: Google hits and 4 months-aggregated calls at the regional level, with post-lockdown interaction

	(1)	(2)	(3)	(4)	(5)	(6)	(7)
				Valid 1522 calls			
	b/se	b/se	b/se	b/se	b/se	b/se	b/se
1522	0.045						
	(0.028)						
Year 2020=1 × 1522	0.139*						
	(0.073)						
Abuse		0.082**					
		(0.031)					
Year 2020=1 × Abuse		0.139**					
		(0.059)					
Feminicide			0.036				
			(0.064)				
Year 2020=1 × Feminicide			-0.245				
			(0.262)				
Rape				-0.043			
				(0.074)			
Year 2020=1 × Rape				-0.177			
				(0.246)			
Domestic violence					0.008		
					(0.026)		
Year 2020=1 × Domestic violence					0.075		
					(0.055)		
Gender-based violence						0.035	
						(0.028)	
Year 2020=1 × Gender-based violence						0.284***	
						(0.065)	
Sexual violence							0.062**
							(0.027)
Year 2020=1 × Sexual violence							0.250**
							(0.103)
Year 2020=1	5.390	6.771**	15.808***	14.382***	11.268***	7.256***	7.103**
	(3.235)	(3.114)	(3.445)	(3.801)	(1.768)	(1.664)	(3.255)
Regional controls	✓	✓	✓	✓	✓	✓	✓
Previous-year dummies	✓	✓	✓	✓	✓	✓	✓
Observations	144	152	152	160	104	136	136

Note: OLS, as indicated. Clustered standard errors at the regional level reported in parentheses. The models follow Eq. 2 in the methods section, yet with additional *Year 2020**keyword interactions (our proxy for the *Post*keyword* interaction with yearly regional-level data). The number of observations varies by model as the availability of yearly queries varies by keyword. Estimated coefficients have been multiplied by 100 to obtain a magnitude that is comparable to the one observed across the other two data sources. * p<0.10, ** p<0.05, *** p<0.01

5 Results using daily data from AREU, Lombardy

Table A12: Google hits and calls to AREU, Lombardy

	AREU calls, Lombardy						
	(1)	(2)	(3)	(4)	(5)	(6)	(7)
1522	0.008<br>(0.025)						
Abuse		0.030<br>(0.027)					
Feminicide			0.076***<br>(0.027)				
Rape				-0.045<br>(0.039)			
Domestic violence					-0.004<br>(0.040)		
Gender-based violence						0.020<br>(0.038)	
Sexual violence							0.047<br>(0.039)
Year FE	✓	✓	✓	✓	✓	✓	✓
Observations	875	875	875	875	875	875	875

Note: OLS, as indicated. Robust standard errors reported in parentheses. The models follow Eq. 1 in the methods section. * p<0.10, ** p<0.05, *** p<0.01.

Table A13: Google hits and calls to AREU, Lombardy, with year and month fixed effects (FE)

	AREU calls, Lombardy						
	(1)	(2)	(3)	(4)	(5)	(6)	(7)
1522	0.009<br>(0.023)						
Abuse		0.000<br>(0.027)					
Feminicide			0.077***<br>(0.028)				
Rape				-0.032<br>(0.040)			
Domestic violence					0.010<br>(0.037)		
Gender-based violence						-0.002<br>(0.037)	
Sexual violence							0.067*<br>(0.038)
Year FE	✓	✓	✓	✓	✓	✓	✓
Month FE	✓	✓	✓	✓	✓	✓	✓
Observations	875	875	875	875	875	875	875

Note: OLS, as indicated. Robust standard errors in parentheses. The models follow Eq. 1 in the methods section. * p<0.10, ** p<0.05, *** p<0.01.

Table A14: Google hits and calls to AREU, Lombardy, with clustering of standard errors (SE) at the month level

	AREU calls, Lombardy						
	(1)	(2)	(3)	(4)	(5)	(6)	(7)
1522	0.008<br>(0.035)						
Abuse		0.030<br>(0.049)					
Feminicide			0.076***<br>(0.023)				
Rape				-0.045<br>(0.058)			
Domestic violence					-0.004<br>(0.027)		
Gender-based violence						0.020<br>(0.038)	
Sexual violence							0.047<br>(0.043)
Constant	88.975***<br>(0.998)	88.718***<br>(1.278)	88.600***<br>(1.014)	89.346***<br>(1.212)	89.004***<br>(0.977)	89.005***<br>(0.978)	88.996***<br>(0.974)
Year FE	✓	✓	✓	✓	✓	✓	✓
Observations	875	875	875	875	875	875	875

Note: OLS, as indicated. Standard errors in parentheses clustered at the month level. The models follow Eq. 1 in the methods section. * p<0.10, ** p<0.05, *** p<0.01.

Table A15: Google hits and calls to AREU, Lombardy, with post-lockdown interaction

	AREU calls, Lombardy						
	(1)	(2)	(3)	(4)	(5)	(6)	(7)
1522	-0.028<br>(0.023)						
Post lockdown=1 x 1522	0.306***<br>(0.055)						
Abuse		-0.001<br>(0.027)					
Post lockdown=1 x Abuse		0.659***<br>(0.211)					
Feminicide			0.059**<br>(0.026)				
Post lockdown=1 x Feminicide			0.095<br>(0.154)				
Rape				-0.050<br>(0.037)			
Post lockdown=1 x Rape				-0.014<br>(0.247)			
Domestic violence					-0.037<br>(0.039)		
Post lockdown=1 x Domestic violence					0.368**<br>(0.187)		
Gender-based violence						-0.008<br>(0.035)	
Post lockdown=1 x Gender-based violence						0.270<br>(0.208)	
Sexual violence							0.007<br>(0.038)
Post lockdown=1 x Sexual violence							0.396**<br>(0.193)
Post lockdown=1	-11.790***<br>(2.662)	-13.358***<br>(3.254)	-9.467***<br>(2.700)	-9.402***<br>(2.878)	-9.360***<br>(2.549)	-9.420***<br>(2.533)	-9.474***<br>(2.527)
Year FE	✓	✓	✓	✓	✓	✓	✓
Observations	875	875	875	875	875	875	875

Note: OLS, as indicated. Robust standard errors reported in parentheses. The models follow Eq. 1 in the methods section, yet with an additional dummy *post* lockdown, alongside *post**keyword interactions. * p<0.10, ** p<0.05, *** p<0.01.

6 Facebook Survey

The Facebook API has been frequently used in the health and social sciences for the purpose of recruiting survey respondents. Facebook's large user base, in fact, enables researchers to implement a swift and demographically diverse recruitment that is representative of the general population once complemented with appropriate post-stratification weights (Ramo & Prochaska, 2012). Facebook is the most widely used online social media platform in Italy, with 30 million 18+ Facebook monthly active users in April 2021. The link to our questionnaire was distributed through an advertisement created through the Facebook Ads Manager. Respondents that clicked the ad were directly enrolled into our subject pool. To minimize self-selection (Lehdonvirta et al., 2021) (e.g., recruiting respondents with unusually greater interest in the Coronavirus or IPV), our ad avoided any mentions to the content of the survey. The survey remained active for three weeks, from February 10 to March 3, 2021. In this time frame, we received 1,131 valid responses. Summary statistics on the main demographic characteristics of the survey respondents are reported in Table A16. Our sample was younger, had a higher proportion of individuals with secondary-degree education, and featured a greater share of women compared to the Italian general population based on the Census (ISTAT, 2020).[26] Regarding the topic of the survey, 67% of respondents declared that they know at least one person who has experienced any form of IPV in their life, while 26% declared that they know at least one person who has experienced any form of IPV in the last year.

Table A16: Summary Statistics for Facebook Survey Respondents

Variable	Mean	Std. Dev.	Min.	Max.
Age	43.98	13.59	18	99
Male	0.231	0.422	0	1
Ph.D.	0.027	0.161	0	1
Master Degree	0.24	0.428	0	1
Bachelor	0.129	0.335	0	1
Diploma	0.477	0.5	0	1
Lower Secondary or below	0.126	0.332	0	1
Observations		1,131		

Figure A3 provides the frequency of words reported by the Facebook survey respondents in the two open-ended questions. As responses to the open-ended ques-

[26] Median age in census = 45.8, median age in the Facebook pool = 43; percentage of the population holding at least a secondary degree in the census = 65%, percentage of the population holding at least a secondary degree in the Facebook pool = 87.6%; percentage of women in the census = 49%, percentage of women in the Facebook pool = 76.9%.

tions included sentences on top of keywords, responses given to both questions were merged and cleaned.[27] Reassuringly, some words that appear most frequently in the word cloud such as *domestic violence* (violenza domestica) and *feminicide* (femminicidio) were already included among the ex-ante keywords employed in the core empirical analyses, thus validating some of our initial choices. Conversely, three frequently cited words that were not included in the ex-ante set of keywords were *anti-violence center* (centro antiviolenza), *woman* (donna) and *help* (aiuto). In the analyses that follow we focus on the former as a unique keyword, while the latter two are combined in no specific order (*woman & help*) given that, if taken individually, they would not be exclusively IPV-related.

Figure A3: Word cloud of keywords provided by Facebook survey respondents when answering the two questions: (i) "Imagine you are seeking information on Google about intimate partner violence. Which keywords would you use and insert in the search engine?" and (ii) "Imagine you are seeking help and support on Google about intimate partner violence. Which keywords would you use and insert in the search engine?"

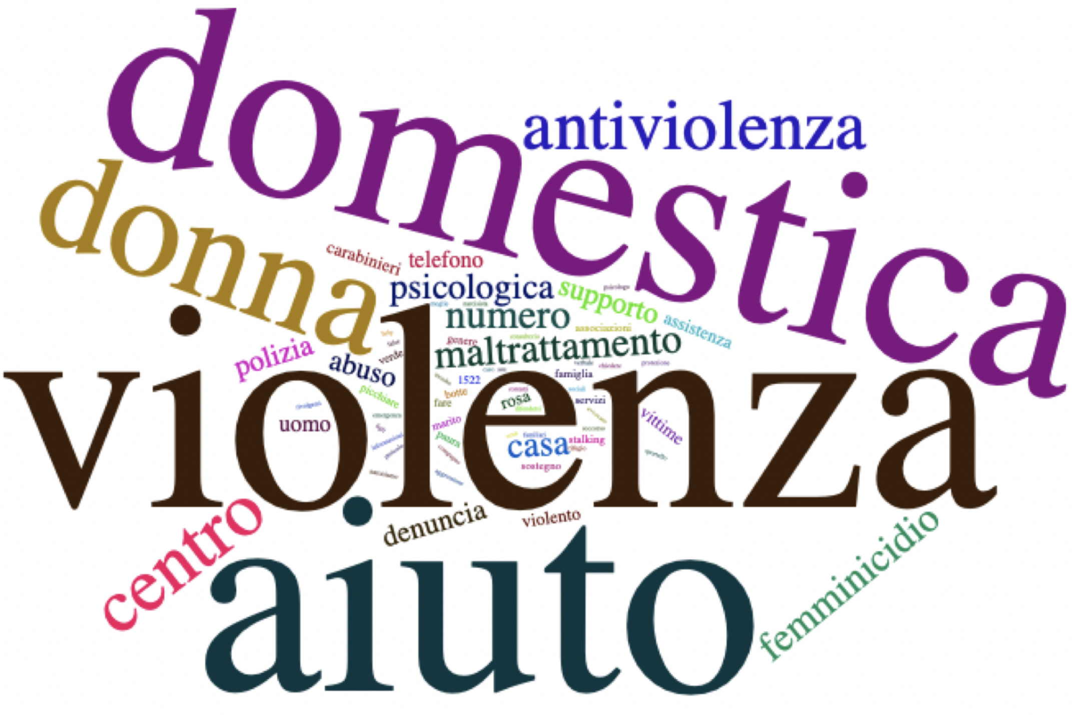

Post-stratification weights applied.

Figure A4 provides instead descriptive statistics on the share of respondents

[27] The text cleaning process involved removing Italian language stop words, removing punctuation, merging plural and singular and masculine and feminine forms of words, as well as merging verbs in infinitive forms based on their root.

that would select a specific keyword listed.[28] This graph refers to the list of 19 keywords, among which respondents could select up to three. The Figure shows that 53% of respondents selected *domestic violence* as the most common keyword — one of the keywords that features most prominently in our core analyses and in the word cloud — followed by *violence & home* (36%), *violence & help* (32%), *violent partner* (31%), *violence & rape* (29%), *violence* (16%), and *abuse* (15%). While *abuse* was also already included in the keywords selected ex-ante, the keywords including "violence" were not. We therefore downloaded them, together with *anti-violence center* and *woman & help* from the word cloud, for the same period under investigation. For some keywords emerging from the histogram, the Google Trends search query provided either partial results for more recent times or no results at all due to low search volumes. We thus only kept the three violence-related keywords with a satisfactory search volume, namely *violent partner*, *violence & help*, and *violence & home*.[29]

Figure A4: Share of individuals choosing selected IPV-related keywords listed by surveyors

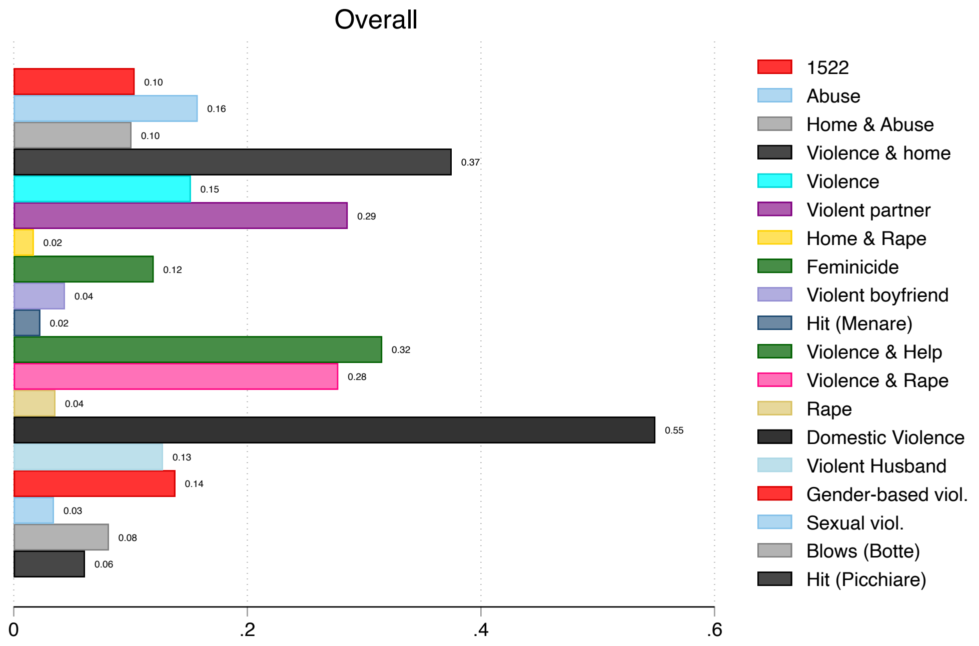

Notes: Post-stratification weights applied. Respondents were offered a list of 19 keywords and they could select up to three. Therefore, shares do not add up to 1.

[28] To account for the demographic biases mentioned above, the bars are weighted by using post-stratification weights by age, gender, and education so as to conform to the Italian census.

[29] For the daily data downloaded at the country level, *violence & rape* and *violent partner* (female) did not produce any result. *Violent partner* (male) and *violence & help* had missing daily data in earlier years but complete weekly data.

Table A17: Cross-correlation table

Variables	1522	Feminicide	Domestic viol.	Gender-based viol.	Sexual viol.	Abuse	Home & Abuse	Home & Rape	Rape	Violent partner	Woman&help	Violence&help	Violence&home	Anti-viol. center
1522	1.000													
Feminicide	0.107	1.000												
Domestic viol.	0.020	-0.001	1.000											
Gender-based viol.	0.051	0.109	-0.013	1.000										
Sexual viol.	0.039	0.000	-0.052	-0.024	1.000									
Abuse	-0.043	0.094	0.003	0.033	0.108	1.000								
Home & Abuse	-0.039	-0.018	-0.059	0.005	-0.011	0.059	1.000							
Home & Rape	-0.029	0.066	-0.018	0.003	0.013	0.008	0.043	1.000						
Rape	0.025	-0.007	0.017	0.009	0.237	0.001	-0.031	-0.012	1.000					
Violent partner	-0.031	-0.052	0.083	0.018	0.032	0.011	0.001	0.026	0.025	1.000				
Woman & help	-0.056	-0.017	0.025	0.041	-0.032	-0.010	-0.072	-0.015	-0.009	0.092	1.000			
Violence & help	0.014	0.037	0.024	0.099	0.024	-0.050	-0.020	-0.004	-0.080	-0.023	0.010	1.000		
Violence & home	0.119	0.035	0.007	0.019	0.014	0.100	0.047	-0.021	-0.058	-0.046	-0.018	-0.032	1.000	
Anti-viol. center	0.055	-0.031	0.052	0.009	0.052	-0.024	-0.028	-0.050	-0.036	-0.011	-0.013	0.045	0.037	1.000

Figure A5: Coefficient plot from regressions of daily 1522 valid calls on Google searches, by keywords emerging from the Facebook survey (whole Italy).

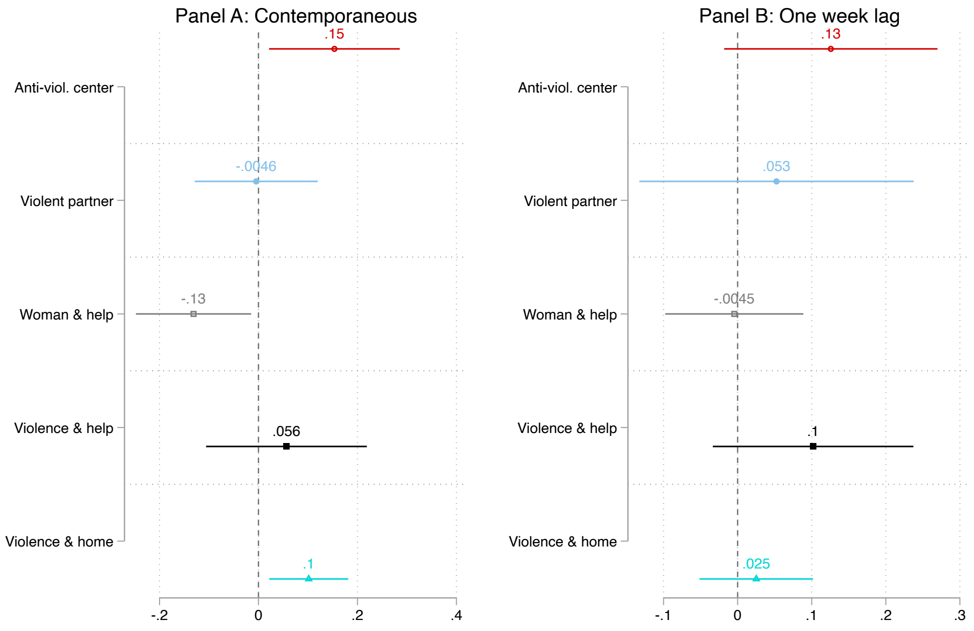

Notes: Data on the outcome from the Equal Opportunity Department, Presidency of Italian Council collected daily from March 1, to June 30, from 2016 to 2020. In Panel A the explanatory variables are contemporaneous the the outcome; in Panel B they are lagged by one week. 95% confidence intervals.
